# Factors associated with C-reactive protein testing when prescribing antibiotics in general practice: a register-based study

**DOI:** 10.1186/s12875-021-01614-6

**Published:** 2022-01-22

**Authors:** Rikke Vognbjerg Sydenham, Malene Plejdrup Hansen, Ulrik Stenz Justesen, Line Bjørnskov Pedersen, Rune Munck Aabenhus, Sonja Wehberg, Dorte Ejg Jarbøl

**Affiliations:** 1 Research Unit of General Practice, Institute of Public Health, University of Southern Denmark, JB Winsløws Vej 9A, 5000 Odense C, Denmark; 2 Center for General Practice at Aalborg University, Aalborg, Denmark; 3 Department of Clinical Microbiology, Odense University Hospital, Odense, Denmark; 4 Danish Centre for Health Economics, Institute of Public Health, University of Southern Denmark, Odense, Denmark; 5 Research Unit for General Practice, University of Copenhagen, Copenhagen, Denmark

**Keywords:** General practice, Anti-bacterial agents, Drug prescriptions, Diagnostic test, C-reactive protein

## Abstract

**Background:**

The use of C-reactive protein (CRP) tests has been shown to safely reduce antibiotic prescribing for acute respiratory tract infections (RTIs). The aim of this study was to explore patient and clinical factors associated with the use of CRP testing when prescribing antibiotics recommended for RTIs.

**Methods:**

A nation-wide retrospective cross-sectional register-based study based on first redeemed antibiotic prescriptions issued to adults in Danish general practice between July 2015 and June 2017. Only antibiotics recommended for treatment of RTIs were included in the analysis (penicillin-V, amoxicillin, co-amoxicillin or roxithromycin/clarithromycin). Logistic regression models were used to estimate odds ratios for patient-related and clinical factors on performing a CRP test in relation to antibiotic prescribing.

**Results:**

A total of 984,149 patients redeemed at least one antibiotic prescription during the two-year period. About half of these prescriptions (49.6%) had an RTI stated as the indication, and a CRP test was performed in relation to 45.2% of these scripts.

Lower odds of having a CRP test performed in relation to an antibiotic prescription was found for patients aged 75 years and above (OR 0.82, 95CI 0.79–0.86), with a Charlson Comorbidity Index of more than one (OR 0.93, 95CI 0.91–0.95), unemployed or on disability pension (OR 0.84, 95CI 0.83–0.85) and immigrants (OR 0.91, 95CI 0.88–0.95) or descendants of immigrants (OR 0.90, 95CI 0.84–0.96). Living with a partner (OR 1.08, 95CI 1.07–1.10), being followed in practice for a chronic condition (OR 1.22, 95CI 1.18–1.26) and having CRP tests performed in the previous year (OR 1.78, 95CI 1.73–1.84) were associated with higher odds of CRP testing in relation to antibiotic prescribing.

**Conclusions:**

Differences were observed in the use of CRP tests among subgroups of patients indicating that both sociodemographic factors and comorbidity influence the decision to use a CRP test in relation to antibiotic prescriptions in general practice. Potentially, this means that the use of CRP tests could be optimised to increase diagnostic certainty and further promote rational prescribing of antibiotics. The rationale behind the observed differences could be further explored in future qualitative studies.

**Supplementary Information:**

The online version contains supplementary material available at 10.1186/s12875-021-01614-6.

## Background

Most acute respiratory tract infections (RTIs) are either non-severe or of viral origin [1–3]. Still, these infections are frequently treated with antibiotics although it will often only add marginal benefits [2, 4, 5].

Refraining from using antibiotics in the mildly to moderately ill patients, where modest benefit can be expected, can minimise the risk of side effects for these patients and in a societal perspective reduce antibiotic resistance and costs [6–8]. This approach will preserve the effectiveness of antibiotics and ensure relevant treatment of serious infections in the severely ill.

Current Danish national guidelines recommend penicillin-V as first-line treatment for RTIs, except for exacerbation of chronic obstructive pulmonary disease (COPD) for which amoxicillin is now recommended. Up until 2017 amoxicillin with clavulanic acid (co-amoxicillin) was recommended for treatment of COPD patients. For patients with penicillin allergy, either clarithromycin or roxithromycin is recommended [9]. In Denmark, azithromycin is not recommended as first line treatment of RTIs in general practice.

General practitioners (GPs) are influenced by many factors when managing patients with signs and symptoms of an infection, and the C-reactive protein (CRP) test can be a useful diagnostic tool when determining if an antibiotic treatment is likely to prove beneficial or should be withheld [10–14].

The vast majority of general practices in Denmark performs CRP tests as point-of-care analysis. Furthermore, all practices can send samples for analysis at the hospital laboratory. Consultations and diagnostic tests are free of charge to the patient and reimbursed from the health authorities [15]. A recent Danish study showed that when GPs are provided with the result of a CRP test it constitutes an important part of the decision to prescribe or withhold antibiotics [16]. Another Danish study found that CRP tests were performed in relation to about 20% of all antibiotic prescriptions issued in general practice, and differences in the use of CRP tests was observed between different antibiotic types [17].

Focussing solely on RTIs, studies have shown that CRP testing can safely reduce the use of antibiotics [18–22]. Consensus exists that CRP testing cannot stand alone but should be interpreted alongside medical history, clinical findings and assessment of risk for the individual patient [23]. The Danish College of General Practitioners encourages the use of CRP as a diagnostic tool when reasonable clinical uncertainty exists. Howeverit is unknown which factors influence GPs to perform a CRP test and whether differences are present in the use among various patient characteristics. The aim of this study was to explore patient and clinical factors associated with the use of CRP testing when prescribing antibiotics recommended for treatment of RTIs. The study can potentially identify areas where the use of CRP tests could be optimised to increase diagnostic certainty and further promote rational prescribing of antibiotics.

## Methods

This nationwide study is a retrospective cross-sectional register-based study linking Danish national registers for the adult population. Data were linked at patient-level using encrypted civil registration numbers.

### Setting

Health services in Denmark are tax-funded and medical expenditures partly subsidised. About 98% of all Danish citizens are registered with a GP. GPs are remunerated through a mixed capitation and fee-for-service system with fees for a consultation and additional fees for performing different services including CRP tests. The out-of-hour services (OOHS) are organised by the GPs in four of the five Danish Regions [15]. In the fifth region the OOHS is organised by the Regional health care service.

### Study population, data sources and variables for the study

*The Danish National Prescription Registry* was used to define the population. This database contains complete information on all prescriptions redeemed by Danish residents at outpatient pharmacies in Denmark from January 1st, 1995 and onwards. The study population comprised all Danish patients aged 18 years and above redeeming prescriptions for one of the following antibiotics: penicillin-V (Anatomical Therapeutic Chemical Classification code (ATC) J01CE02), amoxicillin (ATC J01CA04), co-amoxicillin (ATC J01CR02) or roxithromycin/clarithromycin (ATC J01FA06 and J01FA09) in the project period from July 2015 to June 2017. These antibiotics were selected since they are the recommended as first line treatment for RTIs in Denmark [9]. The two-year timespan was used to take season variation into account, to avoid distortion of data due to e.g. an influenza epidemic and to allow inclusion of patients without frequent antibiotic use. Solely prescriptions issued from general practice or OOHS were included in this study. If a patient had redeemed several antibiotic prescriptions during the project period, only information about the first prescription was used for the analyses. A 14-days antibiotic free-period, covering all types of antibiotics, was required for inclusion in the study. All information was linked using encrypted unique person identification numbers.

In order to define the diagnosis for which each prescription was issued, indication codes were used. We categorised the prescriptions according to indication codes stated on the prescription by the prescriber. A full list of indication codes is available in Additional file 1: List of indication codes and categorisation for prescriptions.

Univariable and multivariable analyses were performed for Model 1) all prescriptions of antibiotics recommended as first line treatment of RTIs and Model 2) the subgroup of these prescriptions with an RTI stated as the specific indication.

The primary outcome measure for the study was the binary variable: whether or not a CRP test was performed in general practice in relation to antibiotic prescriptions. Information about the performance of CRP testing was retrieved from the *Danish National Health Service Register.* Since GPs report their services, including CRP tests, to the *Danish National Health Service Register* at a weekly basis, we defined the date of the CRP test as the Wednesday in the week of reporting. Therefore, a timespan of up to seven days between the performance of a CRP test and redemption of prescription was allowed when defining the two measures as related.

GP reimbursement reports obtained from the *Danish National Health Service Register* were used to compute the number of CRP tests performed and number of consultations in the year before the antibiotic prescription was issued, and whether the patient had follow-up consultations for one or more chronic conditions the previous year. We counted tests and consultations from 14 days before the prescription and one year back to avoid including CRP tests/consultations related to the current event. Season of prescription was defined in two groups: October–March and April–September. The number of antibiotic prescriptions in the previous 365 days before date of first prescription was counted for each patient.

The *Danish National Patient Register* was used to quantify the burden of disease classifying comorbid conditions at the patient level by computing Charlson Comorbidity Indexusing the Quan 2011 method without age adjustment. Healthcare contacts in a 10-year period prior to prescription were used for computing Charlson Comorbidity Index [24].

Socioeconomic information at the patient level were retrieved from *Statistics Denmark* and linked to the prescription by encrypted unique person identification numbers. Socioeconomic variables included information on gender, age groups (18–44, 45–64, 65–74, and + 75 years), length of education (<10 years, 10–15 years, >15 years), labour market affiliation (working, pension, out of workforce/disability pension), cohabitation status (single, married/partner), and ethnicity (Danish, immigrant, descendent of immigrants)*.* For patients with missing information in the year of prescription, we used information from the previous year, alternatively the following year. If neither were available, the patient was assigned to the largest group.

From the *Service Provider Register* we retrieved speciality codes to identify GPs. We used this information to take clustering at practice level into account.

### Statistical analysis

Using descriptive statistics, we generated information on characteristics related to the redeemed antibiotic prescriptions in terms of patient characteristics and clinical characteristics as defined above. We used logistic regression models to estimate odds ratios (OR) with 95% confidence intervals (95CI) for associations between the patient-related and clinical factors and the use of CRP test in relation to prescription of antibiotics for RTIs. Univariable and multivariable analyses were performed for Model 1) all prescriptions of antibiotics recommended as first line treatment of RTIs and Model 2) the subgroup of these prescriptions with an RTI stated as the specific indication. The rationale behind presenting both models is that we wanted to examine use of CRP tests related to RTIs. However, the completeness of the diagnoses stated on the prescriptions was not sufficient to use on its own. To illustrate potential differences when restricting to antibiotics prescriptions with RTI stated as the indication Model 2 was included.

As sensitivity analysis, we also estimated a model including prescriptions with any indications that could possibly contain an RTI, i.e. we included the indications ‘against infection’ and missing indication. Clustering at practice level was taken into account.

All statistical analyses were performed using Stata 16 [25].

## Results

### Characteristics of patients redeeming an antibiotic prescription

Table 1 provides a descriptive overview of the population. A total of 984,149 individuals redeemed a first-time prescription of one of the four selected antibiotic types. An RTI was stated as the indication on about half of the prescriptions (49.6%). Some 12.9% of the scripts did not include an indication and another 17.4% did not specify for which type of infection the antibiotic was issued, and the remaining prescriptions had a specific indication.

**Table 1:** Characteristics of patients redeeming antibiotic prescriptions overall and for the subgroup of prescriptions where the GP stated RTI as indication

	The four types of antibiotics recommended for RTIs	The four types of antibiotics recommended for RTIs with RTI as stated indication
	(n = 984.149)	(n = 487,939)
**Patient characteristics**		
*Gender*		
Male	427,735 (43.5)	203,123 (41.6)
Female	556,414 (56.5)	284,816 (58.4)
*Age*		
18–44	377,404 (38.3)	204,543 (41.9)
45–64	314,242 (31.9)	145,060 (29.7)
65–74	157,136 (16.0)	73,018 (15.0)
75+	135,367 (13.8)	65,318 (13.4)
*Education*		
< 10 years	205,261 (20.9)	102,338 (21.0)
10–15 years	569,126 (57.8)	281,636 (57.7)
> 15 years	209,762 (21.3)	103,965 (21.3)
*Labour market affiliation*		
Working	535,219 (54.4)	275,368 (56.4)
Pension	290,588 (29.5)	138,098 (28.3)
Out of workforce, Disability pension	158,342 (16.1)	74,473 (15.3)
*Cohabitation status*		
Single	505,003 (51.3)	252,247 (51.7)
Married/Partner	479,146 (48.7)	235,692 (48.3)
*Ethnicity*		
Danish	882,056 (89.6)	438,536 (89.9)
Immigrant	90,000 (9.1)	43,266 (8.9)
Descendants	12,093 (1.2)	6137 (1.3)
**Clinical characteristics**		
*Comorbidity - Charlson Index*		
0	806,330 (81.9)	396,184 (81.2)
1	79,086 (8.0)	43,998 (9.0)
> 1	98,733 (10.0)	47,757 (9.8)
*Chronic condition*^a^		
Yes	824,315 (83.8)	409,481 (83.9)
No	159,834 (16.2)	78,458 (16.1)
*Number of consultations previous year*		
0	116,831 (11.9)	57,508 (11.8)
1–4	488,284 (49.6)	244,483 (50.1)
> 4	379,034 (38.5)	185,948 (38.1)
*Number of antibiotic treatments previous year*		
0	737,095 (74.9)	357,337 (73.2)
1	179,624 (18.3)	93,444 (19.2)
> 1	67,430 (6.9)	37,158 (7.6)
*Number of CRP tests previous year*		
0	702,522 (71.4)	338,392 (69.4)
1	185,748 (18.9)	96,543 (19.8)
2-	95,879 (9.7)	53,004 (10.9)
*Prescribing indication*		
Respiratory tract	487,939 (49.6)	487,939 (100.0)
Skin	155,910 (15.8)	–
Other	42,296 (4.3)	–
Infection	170,882 (17.4)	–
Missing	127,122 (12.9)	–
*Season*		
October–March	562,635 (57.2)	308,743 (63.3)
April–September	421,514 (42.8)	179,196 (36.7)

About missing data: Less than 1% were missing for gender, cohabitation, and working status. Around 2% missed information about education. Replaced values are presented in this table.

^a^Defined by patient being followed in practice with at least one chronic condition in the previous year

### Factors associated with the use of CRP

Table 2 presents two multivariable models. Model 1 included all prescriptions of the fourmost commonly recommended antibiotics for treatment of RTIs. A CRP test was performed in relation to 34.1% of these prescriptions. Model 2 was restricted to the subgroup of prescriptions where RTI was stated as the indication. For these a CRP test was conducted in 45.2% of prescribing cases.

**Table 2:** Factors associated with the use of a CRP test in relation to antibiotic prescribing

	Model 1		Model 2	
	Number (%) prescription with CRP test	Multivariable model for the four types of antibiotics recommended for RTIs	Number (%) prescription with CRP test	Multivariable model for the four types of antibiotics recommended for RTIs with RTI as stated indication
	N = 984,149		N = 487,939	
**Patient characteristics**	n (%)	OR (95% CI)	n (%)	OR (95% CI)
Overall	335,755 (34.1)		220,733 (45.2)	
Male	142,507 (33.3)	1	91,759 (45.2)	1
Female	193,248 (34.7)	1.05 (1.04–1.06)	128,974 (45.3)	1.00 (0.98–1.01)
18–44	125,436 (33.2)	1	87,038 (42.6)	1
45–64	108,825 (34.6)	1.02 (1.00–1.03)	69,245 (47.7)	1.19 (1.17–1.22)
65–74	58,595 (37.3)	1.05 (1.01–1.08)	37,339 (51.1)	1.30 (1.24–1.35)
75+	42,899 (31.7)	0.82 (0.79–0.86)	27,111 (41.5)	0.91 (0.86–0.96)
Education <10 years	67,899 (33.1)	1	44,292 (43.3)	1
Education 10–15 years	195,632 (34.4)	1.05 (1.03–1.07)	128,464 (45.6)	1.10 (1.08–1.12)
Education >15 years	72,224 (34.4)	1.04 (1.02–1.07)	47,977 (46.1)	1.13 (1.10–1.17)
Working	185,237 (34.6)	1	124,386 (45.2)	1
Pension	100,956 (34.7)	1.00 (0.98–1.03)	64,294 (46.6)	1.01 (0.97–1.05)
Out of workforce or disability pension	49,562 (31.3)	0.84 (0.83–0.85)	32,053 (43.0)	0.90 (0.88–0.92)
Single	165,734 (32.8)	1	109,632 (43.5)	1
Married/Partner	170,021 (35.5)	1.08 (1.07–1.10)	111,101 (47.1)	1.07 (1.05–1.09)
Danish	303,038 (34.4)	1	199,482 (45.5)	1
Immigrant	28,969 (32.2)	0.91 (0.88–0.95)	18,763 (43.4)	0.92 (0.89–0.96)
Descendant of immigrants	3748 (31.0)	0.90 (0.84–0.96)	2488 (40.5)	0.91 (0.84–0.98)
**Clinical characteristics**				
Charlson 0	274,552 (34.0)	1	180,384 (45.5)	1
Charlson 1	28,911 (36.6)	1.07 (1.05–1.09)	19,668 (44.7)	0.92 (0.90–0.95)
Charlson >1	32,292 (32.7)	0.93 (0.91–0.95)	20,681 (43.3)	0.87 (0.84–0.90)
No chronic conditions	274,124 (33.3)	1	180,549 (44.1)	1
Chronic conditions^a^	61,631 (38.6)	1.22 (1.18–1.26)	40,184 (51.2)	1.24 (1.19–1.29)
0 contacts previous year	36,709 (31.4)	1	24,175 (42.0)	1
1–4 contacts previous year	162,103 (33.2)	0.99 (0.98–1.01)	107,685 (44.0)	1.03 (1.01–1.05)
> 4 contacts previous year	136,943 (36.1)	1.03 (1.00–1.05)	88,873 (47.8)	1.13 (1.10–1.17)
0 antibiotic treatments previous year	253,058 (34.3)	1	165,331 (46.3)	1
1 antibiotic treatment previous year	61,385 (34.2)	0.91 (0.90–0.93)	40,834 (43.7)	0.83 (0.82–0.85)
> 1 antibiotic treatment previous year	21,312 (31.6)	0.75 (0.73–0.77)	14,568 (39.2)	0.64 (0.62–0.66)
0 CRP previous year	222,657 (31.7)	1	146,070 (43.2)	1
1 CRP previous year	70,752 (38.1)	1.33 (1.30–1.36)	46,585 (48.3)	1.23 (1.20–1.27)
> 1 CRP previous year	42,346 (44.2)	1.78 (1.73–1.84)	28,078 (53.0)	1.58 (1.52–1.64)
October–March	210,163 (37.4)	1	141,915 (46.0)	1
April–September	125,592 (29.8)	0.71 (0.70–0.72)	78,818 (44.0)	0.93 (0.92–0.95)

^a^ Defined by patient being followed in practice with at least one chronic condition in the previous year

The multivariable model including all prescriptions within the four types of antibiotics (model 1) showed that females had slightly higher odds of having a CRP test performed in relation to antibiotic prescribing than males (OR 1.05 (95CI 1.04–1.06). Patients aged 75 years and above had lower odds for having a CRP test performed in relation to an antibiotic prescription (OR 0.82 (95CI 0.79–0.86)) compared to the youngest age group (18–44 years). A Charlson Index above 1 was associated with lower odds for CRP testing (OR 0.93 (95CI 0.91–0.95)), compared to Charlson Index 0. Contrary, being followed in general practice for one or more chronic condition showed higher odds for having a CRP test performed in relation to an antibiotic prescription, OR (1.22 (95CI 1.18–1.26). Having an education of less than 10 years, living alone, being out of workforce or at disability pension or being immigrant or descendent from immigrants were all factors associated with lower odds of having a CRP test performed in relation to an antibiotic prescription. The analyses also showed lower odds for CRP testing for patients with more than one antibiotic prescription in the previous year (OR 0.75 (95CI 0.73–0.77), but the opposite if the patient had CRP tests performed in the previous year (OR 1.78 (95CI 1.73–1.84)). Finally, seasonal differences were observed with OR 0.71 (95CI 0.70–0.72) for CRP testing in relation to prescriptions during April to September.

When restricting analyses to prescriptions with a stated RTI (model 2), we found similar trends regarding the variables as for model 1 with a few exceptions. Regarding gender, the model 2 showed no differences between males and females, and for comorbidity a Charlson Index of 1 or above was associated with lower odds of having a CRP test performed in relation to antibiotic prescription.

Results of the univariable models and the sensitivity analysis for prescriptions with a possible RTI indication (comprising prescriptions with RTI, ‘against infection’ or missing indication) are presented in Additional file 2: Additional models. The sensitivity analysis showed no major differences from model 1.

## Discussion

### Main findings

This nationwide population-based study included 984,149 individuals who had received an antibiotic prescription recommended as first line treatment for RTIs during the 2-year study period. A total of 49.6% of the prescriptions had RTI stated as the indication. Differences in the use of CRP tests, when prescribing antibiotics, was identified both in relation to patient and clinical characteristics.

Both models showed that the odds of having a CRP test performed when prescribed antibiotics for an RTI was lower for the elderly (75 years and above), and for patients with comorbidity indicated by the Charlson Comorbidity Index >1. One of the reasons for this finding might be that clinical and medical history and the risk of complications in this age group carry more weight. Hence, GPs may not find CRP testing necessary when deciding to prescribe antibiotics for patients who are more vulnerable due to age and/or comorbidity. Furthermore, a proportion of these patients may be too old or frail to be able to attend general practice and will need home visits with no access to POC tests.

On the other hand, these groups are also more vulnerable to side-effects of antibiotics, meaning that diagnostic certainty should be prioritised. Being followed in general practice for one or more chronic condition was associated with higher odds of having a CRP test performed in relation to an antibiotic prescription for an RTI. In contrast, we find lower odds of CRP testing with higher comorbidity score, indicating that these two measures might assess different aspects. Patients with more than four consultations in practice in the previous year had also higher odds for a CRP test, which may be explained by the fact that these patients are well-known in practice and are more inclined to visit general practice for a test, and perhaps more related to the GP factor than patient factors.

CRP tests are also less used for individuals out of workforce or on disability pension, living alone, immigrants and descendants of immigrants, although the odds are only slightly lower compared to the reference groups. These findings suggest that some groups of patients might be treated differently with no evident differences in terms of risk of complications.

Having more than one CRP test performed in the previous year was a relative strong predictor of having a CRP test performed in relation to an antibiotic prescription, indicating that there is a group of patients, where CRP tests are more often used. Antibiotic treatment in the previous year reduced the chances of having a CRP test performed. This could be well-reasoned, but it could also reflect a tendency of repeating previous treatment without awareness about a potentially new situation. This could indicate that this is an area where CRP tests are not used to the optimal extent.

### Strengths and limitations of the study

This study is based on nationwide registers, recognised for their high validity and ability to cover the entire Danish population, hereby providing a large data material.

However, some limitations must be considered when interpreting the results. The study used the Danish National Prescription Registry which gave us access to redeemed prescriptions. Prescriptions issued, but not redeemed, were not accessible. However, a previous Danish study have found that primary non-adherence for antibiotics is only 6.5% [26].

Another Danish study found that 32% of antibiotic prescriptions had a missing indication [19]. The indications used in this study are the ones stated by the GPs on the prescriptions and only 13% were missing. We attempted to account for this by estimating two models, one with all prescriptions and one with the subgroup of prescriptions with RTI as stated indication. The multivariable models were adjusted for all covariables, since these variables were selected based on hypothesis of influencing the main outcome. We did not take time trends into account, since the study period only covered two years, and as a recent study found that frequency of use of CRP tests did not change over the study period [17].

The database was restricted to patients who redeemed an antibiotic prescription. Hence, we were not able to assess patients for whom the GP chose to perform a CRP test but did not prescribe antibiotics, nor did we have access to the results of the tests. For future studies to further understand the diagnostic process, it could be relevant to study the way GPs use CRP tests also for patients who are not prescribed antibiotics and how the results of the CRP tests are used in the decision-making process. In this study it was not possible to distinguish OOHS from daytime in general practice. Different diagnostic approaches must be expected in general practice and in OOHS, where the physician does not know the patient and has access to fewer diagnostic tools.

The study uses temporal links between CRP tests and antibiotic prescriptions. Data do not confirm whether it is the same GP who performed the test and issued the prescription. In addition, we allowed a timespan of up to 7 days between CRP test and prescription. This approach was necessary since the use of CRP tests are reported on a weekly basis. However, we consider this timespan clinically appropriate and would not expect it to constitute a large limitation to the study.

### Findings in relation to existing knowledge

Antibiotic prescribing can be influenced by many factors such as patients’ medical history; comorbidity; clinical examination; and the result of a CRP test [16, 18, 27]. We found differences in the use of CRP tests among different groups of patients. Odds for using CRP tests were lower for the elderly and patients with comorbidities (defined by Charlson Score above 1). Previous studies have shown that initial clinical judgement carry a high diagnostic value [28, 29]. A recent Danish study found that the CRP threshold for prescribing antibiotics was lowered for patients with higher age and poor general appearance, indicating that CRP plays a minor role compared with these patient groups than with others [30].

Perhaps the clinical judgement plays a larger role with this group of patients, including assessment of probability of benefits of antibiotic use and potential risk of complications to the illness. However, the odds of having a CRP test performed when prescribed antibiotics for an RTI are also lower when the patient is out of workforce or on disability pension, living alone, an immigrant or descendant of immigrants. This correlates with previous studies from other areas showing that there is a social inequality in diagnosing in health care [31, 32]. The results of the present study indicate that GPs are prescribing antibiotics for elderly and socially marginalised using the CRP test as a diagnostic aid to a lesser extent. This could constitute a risk of irrational use of antibiotics including risk of side-effects and selection for resistant bacteria. On the other hand, the differences could also be interpreted as reflecting individualised patient care, thus possibly increasing quality of treatment.

We found that having a CRP performed in the previous year was associated with increased odds of having a CRP test in relation to an antibiotic prescription. This finding is in line with another study, which found that there are differences in the use of diagnostic tests between practices in the range of use of diagnostic tests [33]. Odds ratios cannot necessarily be compared across variables. However, the sizes of the ORs for previous CRP tests and previous antibiotic treatments are quite high, indicating that these somewhat more GP-related factors play a large role. This could be further explored in future studies.

### Implications

This study discovered differences in the use of CRP tests among different patient groups. Socially deprived patients had lower odds of having a CRP test performed in relation to an antibiotic prescription for an RTI. Further studies should attempt to get a deeper knowledge of the rationale behind differences in the use of CRP tests among patients, with a special focus on socioeconomic inequality. The influence of GP factors on the decision to use a CRP test was not assessed in this study, however this angle could also be an important topic for future studies.

Furthermore, the study findings might indicate that the existing evidence on when to use the CRP test could be implemented more explicitly in the clinical recommendations for treatment of RTIs. In a broader perspective, it must be expected that a wider selection of POC tests will be available in the future.

## Conclusion

Differences were observed in the use of CRP tests among subgroups of patients treated with antibiotics for an RTI, indicating that sociodemographic factors and comorbidity influence the decision to use a CRP test in relation to antibiotic prescriptions in general practice.

Potentially, this means that the use of CRP tests could be optimised to increase diagnostic certainty and further promote rational prescribing of antibiotics. The rationale behind the observed differences could be further explored in future qualitative studies.

## Supplementary Information

**Additional file 1: **List of indication codes and categorisation for prescriptions.

**Additional file 2: **Additional models.

## Data Availability

The data that support the findings of this study are available from Statistics Denmark, but restrictions apply to the availability of these data, which were used under license for the current study, and so are not publicly available. Data are however available from the authors upon reasonable request and with permission of Statistics Denmark.

## Abbreviations

CRP: C-reactive protein
95CI: 95% confidence interval
GP: General practitioner
OR: Odds ratio
RTI: Respiratory tract infection
