# Supplementary material for: Factors associated with C-reactive protein testing when prescribing antibiotics in general practice: a register-based study
Source: BMC Prim Care. 2022 Jan 22;23:17. doi: 10.1186/s12875-021-01614-6 (PMC8783519; doi:10.1186/s12875-021-01614-6)
Supplement: Supplementary file 1 — Additional file 1: List of indication codes and categorisation for prescriptions. [file 12875_2021_1614_MOESM1_ESM.pdf]

Prescribing antibiotics: Factors associated with C-reactive protein testing in general practice. A register-based study

Authors: Rikke Vognbjerg Sydenham[1], Malene Plejdrup Hansen[2], Ulrik Stenz Justesen[3], Line Bjørnskov Pedersen[1,4], Rune Munck Aabenhus[5], Sonja Wehberg[1], Dorte Ejg Jarbøl[1]

Affiliations:

[1]Research Unit of General Practice, Institute of Public Health, University of Southern Denmark, Denmark

[2]Center for General Practice at Aalborg University, Aalborg, Denmark

[3]Department of Clinical Microbiology, Odense University Hospital, Denmark

[4]Danish Centre for Health Economics, Institute of Public Health, University of Southern Denmark, Denmark

[5] Research Unit for General Practice, University of Copenhagen, Denmark

***Corresponding author:**

Rikke Vognbjerg Sydenham, Research Unit of General Practice, Institute of Public Health, University of Southern Denmark, JB Winsløws Vej 9A, 5000 Odense C, Denmark, Email: rsydenham@health.sdu.dk

Appendix 1

List of indication codes and categorisation for prescriptions

Indication category	Indication code	Indication stated on the prescription
RTI	0000121	against otitis media
	0000122	against sore throat
	0000123	against pneumonia
	0000127	against bronchitis
	0000192	against inflammation of the ear canal
	0000197	against sinusitis
	0000332	against lung infection
	0000379	in chronic obstructive pulmonary disease (COPD)
	0000396	bronchitis with bronchospasm
	0000462	against whooping cough
	0000463	against scarlet fever
	0000464	against the exacerbation of chronic obstructive pulmonary disease (COPD)
	0000481	against epiglottitis
	0000665	against empyema
	0000709	against ear, nose, throat infection
	0000740	acute exacerbation of chronic bronchitis
	0000771	acute exacerbations of chronic bronchitis
Skin infections	0000128	against impure skin
	0000224	against infection of the skin
	0000230	against infected eczema
	0000231	against psoriasis with infection
	0000234	against acne
	0000360	against inflammation of the skin

	0000417	against skin and soft tissue infection
	0000455	prophylaxis after animal or human bites
	0000457	against childhood ulcers
	0000458	against wound infection
	0000459	against erysipelas
	0000714	against rosacea
Other	0000003	against stomach ulcers
	0000006	against acid reflux
	0000021	for intestinal disinfection
	0000024	against inflammation of the intestine
	0000026	against diarrhoea
	0000048	for heart disease
	0000069	against heavy bleeding
	0000079	against elevated cholesterol
	0000085	against yeast infection
	0000094	Birth control pills
	0000096	hormone supplementation
	0000103	against cystitis
	0000104	against urinary tract infection
	0000113	against hay fever
	0000143	against muscle pain
	0000144	pain reliever
	0000145	against pain
	0000168	against depression
	0000186	against eye disease
	0000199	against runny nose
	0000202	mod asthma
	0000203	for the prevention of asthma
	0000205	expectorant
	0000206	against cough
	0000207	against dry cough
	0000211	against athlete's foot
	0000222	against eczema
	0000225	against genital warts
	0000227	against skin disorder
	0000249	against inflammation of the colon
	0000265	for the prevention of urinary tract infection
	0000298	against eye inflammation
	0000325	in lung disease
	0000367	against intestinal disease
	0000412	treatment of neutropenia by chemotherapy
	0000425	against Lyme disease, stages II and III
	0000427	against bacterial infection in bones and joints
	0000434	for the prevention of yellow fever
	0000444	against inflammation of the abdominal cavity
	0000445	against inflammation of the abdomen
	0000446	against inflammation of the epididymis
	0000447	against inflammation of the urethra
	0000448	against pelvic inflammatory disease
	0000449	against inflammation of the bladder neck gland
	0000450	against inflammation of the vagina
	0000451	against Chlamydia / mycoplasma infection
	0000453	against syphilis
	0000454	against Borrelia infection
	0000456	against mastitis
	0000460	against heart valve inflammation
	0000461	for prevention of heart valve inflammation
	0000465	against peptic ulcer (Eradication of Helicobacter pylori)
	0000483	against intra-abdominal infection

	0000484	for prophylaxis before surgery
	0000486	for prevention of serious infection
	0000524	against toothache
	0000541	against neuropathic pain
	0000672	preventive against cardiovascular disease
	0000710	against tooth, mouth, jaw infection
	0000719	preventive in cardiovascular disease
	0000720	against Lyme disease, stage I
Against infection	0000084	against infection
	0000312	against inflammation
