# Supplementary material for: Factors associated with C-reactive protein testing when prescribing antibiotics in general practice: a register-based study
Source: BMC Prim Care. 2022 Jan 22;23:17. doi: 10.1186/s12875-021-01614-6 (PMC8783519; doi:10.1186/s12875-021-01614-6)
Supplement: Supplementary file 2 — Additional file 2: Additional models. [file 12875_2021_1614_MOESM2_ESM.pdf]

Prescribing antibiotics: Factors associated with C-reactive protein testing in general practice. A register-based study

Authors: Rikke Vognbjerg Sydenham[1], Malene Plejdrup Hansen[2], Ulrik Stenz Justesen[3], Line Bjørnskov Pedersen[1,4], Rune Munck Aabenhus[5], Sonja Wehberg[1], Dorte Ejg Jarbøl[1]

Affiliations:

[1]Research Unit of General Practice, Institute of Public Health, University of Southern Denmark, Denmark

[2]Center for General Practice at Aalborg University, Aalborg, Denmark

[3]Department of Clinical Microbiology, Odense University Hospital, Denmark

[4]Danish Centre for Health Economics, Institute of Public Health, University of Southern Denmark, Denmark

[5] Research Unit for General Practice, University of Copenhagen, Denmark

***Corresponding author:**

Rikke Vognbjerg Sydenham, Research Unit of General Practice, Institute of Public Health, University of Southern Denmark, JB Winsløws Vej 9A, 5000 Odense C, Denmark, Email: rsydenham@health.sdu.dk

Appendix 2

Additional models:

	Univariable model for the four types of antibiotics recommended for RTIs (N = 984,149)	Univariable model for the four types of antibiotics recommended for RTIs with *RTI* as stated indication (N = 487,939)	Univariable model for the four types of antibiotics recommended for RTIs with *RTI* or *against infection* as stated indication or *missing indication* (N = 785,943)	Multivariable model for the four types of antibiotics recommended for RTIs with *RTI* or *against infection* as stated indication or *missing indication* (N = 785,943)
	OR (95CI)	OR (95CI)	OR (95CI)	OR (95CI)
Patient characteristics				
Male	1	1	1	1
Female	1.07 (1.05-1.08)	1.00 (0.99-1.02)	1.04 (1.03-1.05)	1.03 (1.02-1.04)
18-44	1	1	1	1
45-64	1.06 (1.05-1.08)	1.23 (1.20-1.26)	1.10 (1.08-1.12)	1.06 (1.04-1.08)
65-74	1.19 (1.17-1.22)	1.41 (1.37-1.46)	1.24 (1.21-1.27)	1.09 (1.06-1.13)
75+	0.93 (0.90-0.96)	0.96 (0.91-1.01)	0.93 (0.90-0.97)	0.84 (0.80-0.87)
Education < 10 years	1	1	1	1
Education 10-15 years	1.06 (1.04-1.08)	1.10 (1.08-1.12)	1.08 (1.06-1.10)	1.07 (1.05-1.08)
Education > 15 years	1.06 (1.03-1.09)	1.12 (1.09-1.16)	1.09 (1.06-1.12)	1.07 (1.04-1.10)

Working	1	1	1	1
Pension	1.01 (0.98-1.03)	1.06 (1.02-1.09)	1.01 (0.99-1.04)	1.00 (0.98-1.03)
Out of workforce or disability pension	0.86 (0.85-0.88)	0.92 (0.90-0.94)	0.86 (0.85-0.88)	0.85 (0.83-0.86)
Single	1	1	1	1
Married/Partner	1.13 (1.11-1.14)	1.16 (1.14-1.18)	1.14 (1.12-1.16)	1.09 (1.07-1.10)
Danish Ethnicity	1	1	1	1
Immigrants	0.91 (0.87-0.94)	0.92 (0.88-0.96)	0.89 (0.86-0.94)	0.90 (0.86-0.94)
Descendants of immigrants	0.86 (0.80-0.92)	0.82 (0.76-0.88)	0.84 (0.78-0.90)	0.89 (0.83-0.96)
Clinical characteristics				
Charlson 0	1	1	1	1
Charlson 1	1.12 (1.09-1.14)	0.97 (0.94-0.99)	1.04 (1.01-1.06)	1.01 (0.98-1.03)
Charlson >1	0.94 (0.92-0.97)	0.91 (0.88-0.95)	0.92 (0.90-0.95)	0.91 (0.89-0.93)
No chronic conditions	1	1	1	1
Chronic conditions	1.26 (1.23-1.29)	1.33 (1.29-1.38)	1.10 (1.08-1.12)	1.02 (1.00-1.03)
0 contacts previous year	1	1	1.26 (1.23-1.29)	1.08 (1.05-1.11)
1-4 contacts previous year	1.08 (1.07-1.10)	1.09 (1.06-1.11)	1	1
>4 contacts previous year	1.23 (1.21-1.26)	1.26 (1.23-1.30)	1.28 (1.24-1.32)	1.23 (1.19-1.27)
0 antibiotic treatments previous year	1	1	1	1
1 antibiotic treatment in previous year	0.99 (0.98-1.01)	0.90 (0.89-0.92)	0.94 (0.92-0.95)	0.86 (0.85-0.88)
>1 antibiotic treatment in previous year	0.88 (0.86-0.91)	0.75 (0.73-0.77)	0.80 (0.78-0.82)	0.68 (0.66-0.70)
0 CRP previous year	1	1	1	1
1 CRP previous year	1.33 (1.30-1.35)	1.23 (1.20-1.26)	1.31 (1.28-1.34)	1.32 (1.29-1.35)
>1 CRP previous year	1.70 (1.66-1.75)	1.48 (1.44-1.53)	1.64 (1.59-1.69)	1.75 (1.69-1.81)
October-March	1	1	1	1
April-September	0.71 (0.70-0.72)	0.92 (0.91-0.94)	0.80 (0.79-0.81)	0.80 (0.79-0.81)
